# Determination of Compressive Stress Limits for Tightened Plastic Components up to 3 mm in Bolted Joints Applicable in the Automotive Industry

**DOI:** 10.3390/polym17030268

**Published:** 2025-01-21

**Authors:** Zuzana Murčinková, Rudolf Holíček, Petr Baron, Martin Onufer

**Affiliations:** Faculty of Manufacturing Technologies, Technical University of Košice, 080 01 Prešov, Slovakia; zuzana.murcinkova@tuke.sk (Z.M.); rudolf.holicek@student.tuke.sk (R.H.); martinonufer@hotmail.sk (M.O.)

**Keywords:** bolted joint, plastic materials, compressive stress, limit value, plastic deformation, automotive industry

## Abstract

This paper addresses the analysis of compressive stress limit values of plastic components with a thickness of no more than 3 mm used in bolted joints, especially in the automotive industry. The results of the compression tests show that the compressive stress limit values often exceed the tensile stress limit values specified in the material data sheets, which has a significant impact on the way in which reliable bolted joints are designed without the risk of plastic deformation. In addition to compression tests, stress tests involving axial force and torque (combined load typical for bolted joints) were also performed. Th results of both types of tests were compared in the final table, involving a comparison of yield strength under compression and yield strength under a combined load with yield strength and/or stress at break from material data sheets, estimated using tensile stress tests. Various plastic materials were tested, including Acrylonitrile Butadiene Styrene (ABS), Polyamide (PA), Polyoxymethylene (POM), Polypropylen (PP) and the glass fiber-reinforced materials. The tests showed that it is possible to exceed the tensile stress limit in material data sheets by 5 to 10% without plastic deformation and by approximately 50%, in some cases by 280%, when loading by pure compression. Considering the combined load, the compressive stress limit values are within the range of 95 to 224% of tensile stress limits. The results of the study contribute to the optimization of the plastic tightened components design and reduce the need for excessive testing in automotive production.

## 1. Introduction

Bolted joints are one of the most widespread means of joining components in the automotive industry. For this type of joints, materials that meet the requirements for strength, durability and corrosion resistance are most often used in the automotive industry; plus, they must be able to handle various types of loads. The dominant material used for bolted joints in the automotive industry is steel, including low-carbon, alloyed and high-strength steel. Steel is popular for its high strength, low cost and wide availability. Steel bolts are designed to withstand high stress and are typically used in critical areas such as engines, chassis and vehicle structural elements. Non-ferrous metals such as aluminum, titanium, magnesium or copper alloys are becoming increasingly popular with bolted joints applied in the automotive industry. Aluminum is increasingly used in the automotive industry, especially to reduce vehicle weight and improve fuel efficiency. Aluminum bolts are often used in applications where the weight and corrosion resistance requirements are important. The proportion of aluminum bolts in the automotive industry is smaller than that of steel bolts, but it is growing, especially in electric and hybrid vehicles. Their disadvantage is the toughness of the material and, from a production point of view, the price, which is usually higher than the price of steel bolts. Magnesium alloys are attractive for applications where a light weight is important, because magnesium is one of the lightest structural metals. Such bolts may be present in applications requiring good electrical conductivity (e.g., electric vehicles) or in joints with sufficient corrosion resistance. Magnesium alloys are used in specific cases where weight reduction is needed—for example, in aerospace, automotive or electronics manufacturing. However, these applications are limited to cases where high-strength bolts are not needed and joints are not subjected to high mechanical stress. Bolts and fasteners are more often made of lighter aluminum or titanium alloys, which provide a better compromise between strength, weight and corrosion resistance. Bolted joints made of copper and its alloys are less common.

In recent years, with an increasing emphasis on reducing weight and improving efficiency, plastic materials have been increasingly used in the automotive industry as a bolted connections material. Plastic bolted joints are mainly used in interiors such as panels, seats and other parts where the emphasis is on weight reduction. In some cases, plastic joints are also used for exterior parts such as covers and components, where, in addition to weight minimization, corrosion resistance is an important parameter. Plastic bolts are mostly used as so-called plastic tapping screws (bolts that form their own thread, e.g., in a plastic or in the seat foam). For bolts with a metric thread, plastic is not used, mainly due to high thermal expansion and subsequent high relaxation, which can lead to the joint loosening. Plastic bolted joints contribute to reducing the weight of a vehicle. This is crucial for improving fuel efficiency. They are also resistant to corrosion and chemicals. This property is important for the long service life of joints. Plastic can be easily molded into different shapes and designs. However, plastic materials usually do not have strength as high as steel bolts do. This may limit their use in heavy duty applications. It should also be remembered that plastic may be subject to creep and deformation at higher temperatures, which adversely affects the stability of the bolted joint.

Plastic material connected by bolted joints has greater representation in the automotive industry than plastic fasteners do (bolts, rivets, etc.). Materials such as polyamides (PA), polypropylene (PP), polyoxymethylene (POM), acrylonitrile butadiene styrene (ABS), PA6 and PA66 are commonly used. These materials provide good strength and resistance to creep. PP is used for bolted joints that do not require a high strength but are suitable for applications where a light weight and a low cost are important. POM is popular for its excellent mechanical properties and stability. It is suitable wherever precision and wear resistance are required. ABS is known for its strength and impact resistance, which predestines it for use in applications that require mechanical properties.

As sustainability and weight reduction become more important, the use of plastics in the automotive industry is expected to continue growing, extending beyond just bolted joints. Yet, despite the aforementioned advantages, the mechanical properties of plastic materials, such as their ability to transfer stress in bolted joints, have been explored less than traditional metal materials. It is especially important to set limit values for the compressive stress that these materials can transfer without plastic deformation. It should also be remembered that plastics are sensitive to temperature changes and can absorb moisture from the environment. These environmental factors can change the mechanical, physical and chemical properties of plastics, thus affecting their performance and durability in various applications.

This research, which is the subject matter of the study conducted, focused on determining the compressive stress limit values of plastic components with a thickness of up to 3 mm in bolted joints using steel bolts. The study used various plastic materials, including ABS, PA, PBT, POM and PP, as well as glass fiber-reinforced materials. The goal was achieved by compressive testing using a universal testing machine loaded with pure compressive axial force and a combination of axial force and torque to efficiently and economically design bolted joints without excessive testing. The results of the present study, dealing with bolted joints used for fastening plastic components, can provide practical guidelines for the design of plastic bolted joints in the automotive industry.

## 2. Overview of the Papers Published in the Field of the Issue Addressed

As part of the stage of analysis of the issue addressed, the authors of this study conducted a review of the published literature dealing with similar issues. Several scientific studies researching the application of bolted joints in combination with plastic material exist, dealing in particular with various aspects of their strength and durability. For example, Ref. [1] describes the suitability of basic materials for vehicle construction. Low-carbon steel and cast iron are being replaced by materials with higher specific strength and rigidity: advanced high-strength steels, aluminum, magnesium and polymer composites. The key challenge is to reduce the cost of manufacturing structures with these new materials. Maximizing weight reduction requires optimized designs using multiple materials in different forms. In [2], research into the behavior of a bolted joint installed on a tubular plastic lug with supporting ribs using finite element analysis (FEA) and other experiments is presented. When connecting the components of the interior of a commercial vehicle, most plastic boards are mounted on the ribs of the lug for easier assembly, unlike conventional plastic joints, so that the ribs can withstand the prestress of the bolt rather than that of the lug itself. For a lug with relatively weak ribs, the clamping force did not increase above a certain value, although the tightening torque gradually increased during the fastening process. The relationships between tightening torque, clamping force and bolt rotation angle were investigated using the FEA for nine different lugs with ribs of different shapes. Ref. [3] comprises a study on joining polyetherimide (PEI) polymer parts reinforced with glass fibers, which are widely used in the automotive industry. The study provides knowledge of the processes for joining self-tapping screws with polymeric materials of a mechanical nature. The article in [4] describes a study of the strength of a threaded joint made of a reinforced composite material based on the simulation of the stress-deformation state of the joint, using the finite element method and a subsequent experimental verification of the results of the tests carried out. The conclusions confirm that threaded joints of parts made of fiber-reinforced composite materials are specific to manufacturing. The analysis revealed that the combination of the new processing technology and the innovative geometry of the threaded joint makes it possible to achieve significantly higher final tensile strength compared to standard metric threads produced directly in the parts, and even compared to thread inserts. Research in the field of joining different types of materials has been published in [5]. This paper summarizes and evaluates state-of-the-art research on combining different materials. Current and emerging joining technologies are assessed according to their mechanisms of joint formation, i.e., mechanical, chemical, thermal or hybrid processes. The paper describes the methods for selecting certain processes and summarizes future challenges in research on the joining of different materials. The paper [6] is devoted to the issue of normalized tightening of threaded joints in the process of ultrasonic assembly. Tightening options are discussed based on the system’s response to the impact acting on the threaded joint during the tightening process. A tightening torque estimation method is considered for achieving the yield strength of the material. In [7], experimental and theoretical research on the resistance of threaded joints to low-cycle and multi-cycle stress is described. It turns out that the existing calculation methodologies are not sufficiently valid and, therefore, the existing methods of investigation have been improved upon, taking into account structural, technological and operational factors using decay mechanics criteria and shakedown theories. The paper in [8] describes the design of and effective method for preparing bolted laminates from CFRP (carbon fiber-reinforced plastic). Static tensile tests of structures connected by CFRP bolts were performed under various conditions including the number of bolts, the torque and the temperature. The results of the analysis indicate that the bolted CFRP structures suffered mainly partial shear extrusion failure at a low temperature and at room temperature. The conducted analysis provides reference information for researchers in the field of composite bolted joint design and reveals the influence of torque and bolt tightening temperature on the mechanical properties of a CFRP bolted joint. The effects of the clearance of the bolt hole on the stiffness and strength of composite bolted joints are described in [9]. The maximum efficiency of joints in composite structures is usually lower than in metals, so poorly designed joints significantly reduce the weight advantage of composites over metals. In a typical production environment, the diameter of fasteners and holes varies within certain permissible tolerances. The combination of bolt tolerances may result in a number of permitted bolt hole alignments, which are generally clearance rather than fastener alignments in composites, due to concerns about damage to the composite during the insertion of the fastener and also the possible removal of the fastener during inspections. The published scientific and professional papers [10,11,12,13,14] emphasize the need to optimize bolted joints for composite materials, due to their susceptibility to high stress and fatigue damage. The mechanical failure of composite joints is a frequent subject of research, especially in applications where composite materials are combined with metals. These studies are relevant to industries such as automotive and aerospace, where composite materials are increasingly used to reduce weight and improve performance. These publications offer important insights for the development and optimization of composite bolted joints.

## 3. Key Aspects of the Addressed Topic

The primary objective of the conducted research and study focused on the design and optimization of bolted joints of plastic components in the automotive industry. It was important to identify the compressive stress limits of plastic materials at which no plastic deformation of up to 3 mm-thick tightened components occurs. At the same time, the behavior of plastic materials under axial force and torque combination was addressed, which aimed at achieving an effective design of bolted joints without excessive costs of further testing. The mechanical properties of various plastic materials, such as polyamides, polypropylene, polyoxymethylene and plastics reinforced with glass fibers, were also compared. The aim was to determine the optimal dimensions of the bolted joints with respect to these limits and to ensure that the joints transfer the required stress without suffering subsequent damage.

An essential part of the joint design is the determination of the correct dimensions of the bolt head and bearing surface so that the stress under the bolt head is within the limit within which the plastic material can transfer that stress without plastic deformation occurring. This includes the following:

- defining the diameter of the bolt head bearing surface;
- selecting the optimal tightening torque that transfers the axial force to the plastic component without damaging it;
- determining the thickness and properties of the plastic component, while ensuring the thickness of the material does not exceed 3 mm (the standard thicknesses of materials used in the automotive industry).

For effective joint design, tests were carried out to determine the compressive limit stress values for different materials at pure axial force and at torque, which was converted into axial force (weld force). By testing, we wanted to verify how plastic materials behave under different types of stress and determine their ultimate strength, so as to design joints that would be reliable and, at the same time, effective in terms of production costs. When using plastics in the automotive industry, factors such as creep, which is a slow plastic deformation caused by prolonged exposure to stress, must be considered. This deformation may weaken the joint over time and lead to its failure. Therefore, it is important to determine not only the initial stress that the plastic can handle but also its dynamic resistance to the long-term effects of external forces.

Another important aspect of the addressed issue is the cost-effectiveness of the implemented design. Using optimal design procedures can avoid unnecessary costs for tools and molds that would otherwise be needed for validation and testing. The effective design of bolted joints based on precise limits of the mechanical properties of plastic materials thus contributes to reducing the time needed for the development of new components.

The addressed issue points to the complexity of the design and testing of plastic joints and the importance of accurately determining the limit values of the combination of stress and torsion to achieve reliability and cost savings in automotive manufacturing.

### Materials for Research and Methods of Conducted Experiments

The EN ISO 6892-1 standard [15] defines yield strength as the point at which a material begins to show signs of permanent (plastic) deformation while maintaining a constant or only slightly increasing axial force. This point represents the transition of the material from elastic behavior, where the deformation returns to its original state after relief, to the creep area, where the deformation remains even after the material is freed from stress. When plastic deformation emerges without a further increase in stress, it means that the material has reached its yield point. This standard also defines ultimate strength, which is the maximum force achieved during stress application. The yield strength definition makes it clear that, after exceeding this point, plastic and, thus, permanent deformation of the stressed component takes place. If we stress the material with tension or pressure and do not exceed the yield strength, the material deforms elastically, which means that, upon relief, it returns to its original state without breaking or suffering permanent deformation. Exceeding the yield strength is, in most cases, not acceptable for several reasons. The main reason is deformation of the joined material, the bolt or the nut. Visible deformations on the component that is joined are not acceptable from either the visual or functional point of view.

In the case of plastic components, we can expect two types of material creep. For soft materials such as PP T20 (reinforced 20% talc polypropylene), slow deformation at low axial forces can be observed. This means that the material will be easily deformable. For soft materials, the plastic deformation area is high. The material will be stretched or compressed to a high degree, while cracking is unlikely with materials this soft. Plastic materials with higher yield strength and ultimate strength, such as PA GF40 (PA—polyamide, GF40—40% glass fibers), may show cracking due to high stress. The reason is that the area between the beginning of plastic deformation and the cracking of the material is relatively small compared to soft materials. Neither cause of these failures is acceptable; therefore, it is important to establish the maximum axial force that the plastic material will transfer without breaking or suffering deformation.

If the torque was higher when tightening the bolt than the material yield strength, the material would begin to be subject to plastic deformation, i.e., “creep”. This means that the stress in the contact surfaces exceeds the tolerated capacity of the material, which leads to its deformation. This deformation is visually observable in the form of imprints, which are known as “dents”. In some cases, white blemishes may appear on the surface of the material, the so-called “white spots”, which signal overload. If the material was stressed to the maximum, but the ultimate strength was not reached, the material would relax over time. A characteristic typical for this phenomenon is a gradual reduction in the axial force in the joint, which is a natural effect in bolted joints, regardless of whether plastic or steel components are used. However, to minimize this phenomenon, it is important to choose the right design solutions [16].

There are several approaches to reducing the risk of plastic deformation and plastic material relaxation. The key is to accurately identify the behavior of a particular plastic material during tightening. Mechanical properties, composition, the presence of reinforcements and the conditions in which the material will be used play an important role. However, the presence and orientation of glass fibers is often difficult to determine accurately. If the axial force acts in the glass fibers’ direction, the maximum axial strength will be higher. Conversely, when oriented perpendicularly to the glass fibers, the material may exhibit lower ultimate strength values. This is especially true for polyamide, which can absorb water, thereby increasing its toughness [13,16,17].

The key principle in the design of bolted joints is to reduce the local stress to the least value possible. This goal can be achieved in a variety of ways, including reducing the torque, using adhesives, enlarging the bolt bearing area, adding flat washers or special washers such as NORD LOCK, or selecting higher strength plastics [18].

From an economic point of view, the use of bolts with a larger head diameter proved to be the most effective in practice. The minimum diameter of the bearing surface must be determined on the basis of the mechanical properties of the particular plastic material, in order to ensure an optimal balance between strength and deformation properties.

The diameter of the bearing surface is determined so that the stress on the bearing surface is not greater than the permissible stress value. First of all, it is necessary to determine the tightening torque for the bolt diameter. The tightening torque is derived from Equation (1), based on the standard VDI 2230-1, “Systematic calculation of highly stressed bolted joints—Joints with one cylindrical bolt” [19].

(1) $$M_{A}=F_{M}\left[0.16*P+0.58*d_{2}*{µ}_{Gmin}+\frac{D_{Km}}{2}{µ}_{Kmin}\right]$$

where *M*_A_—tightening torque, *F*_M_—preload when tightening, *P*—thread pitch, *d*_2_—pitch diameter of the thread, µ_Gmin_—friction coefficient on the threaded part, *D*_Km_—mean diameter of the bearing surface and µ_Kmin_—friction coefficient on the bearing surface.

If the tightening torque is within the interval defined by the customer or internal regulations, the stress value on the bearing surface is checked. This is calculated using Equation (2) [19], as follows:

(2) $$p_{M}=\frac{F_{M}}{A_{P}}$$

where *p*_M_—bearing stress on the bearing surface, *F*_M_—preload axial force and *A*_P_—bearing area (perpendicular to *F*_M_).

The calculated bearing stress p_M_ must be less than the compressive limit stress value that the material is able to withstand. Otherwise, the bolt head diameter must be increased. For this reason, both the value *D*_Km_ and the value *A*_P_ will increase. While the increased value *D*_Km_ increases the tightening torque value because more friction has to be overcome, the increased value *A*_P_ reduces the bearing stress value on the bearing surface.

However, such a solution may cause a problem in terms of the bolt manufacturability. Standard large head bolts have a bolt head diameter of approximately 2.3 times the diameter of the bolt body. However, in some cases, these head diameters are not sufficient and it is necessary to define the head diameter at 2.75 times the bolt body diameter or larger. This often causes a problem with the bolt’s manufacturability at the quality required. The production of such bolts is also more costly.

In the experimental part of our study, tests were carried out on plastic components that were stressed with a compressive axial force. The testing consisted of two main types of tests. The first test measured the deformation of plastic materials under compressive stress using the Instron universal testing machine 5982 Series, Instron, Canton, MA, USA. The second test focused on a combination of the compressive axial force and torque when tightening the bolted joint to specify an appropriate tightening torque.

As part of the tests, 18 different material samples commonly used in the automotive industry were used, including polyamides (PA), polypropylenes (PP), polyoxymethylene (POM), acrylonitrile butadiene styrene (ABS) and polybutylene terephthalate (PBT). Glass fiber-reinforced composite materials, such as PA6 GF30, Celanyl, Trebatice, Slovakia and POM GF10, Celanese, Dallas, TX, USA which have improved mechanical properties, were also included in the materials tested. For the purposes of testing, material samples were provided by companies engaged in the production and development of plastic components for the automotive industry. The test results allowed for a detailed comparison of mechanical behavior of different classes of plastic materials and can provide valuable data for the design of reliable and efficient bolted joints.

## 4. Testing of Material Samples—Compressive Stress Test

The compressive stress test was performed using the Instron Universal Testing Machine 5982 Series. The compressive stress test was carried out to analyze the behavior of the material under compressive stress without the influence of other types of stress, such as bending or shear. This test is crucial for understanding the material’s response to direct stress, which is essential for applications where the material is subjected exclusively to compressive stress. The main objective was to determine the compressive stress limits at which elastic deformation of the material occurs. The compressive stress test was designed to simulate the conditions of using a bolt with a washer where the minimum torque tension is on the bearing surface.

**Figure 1:**
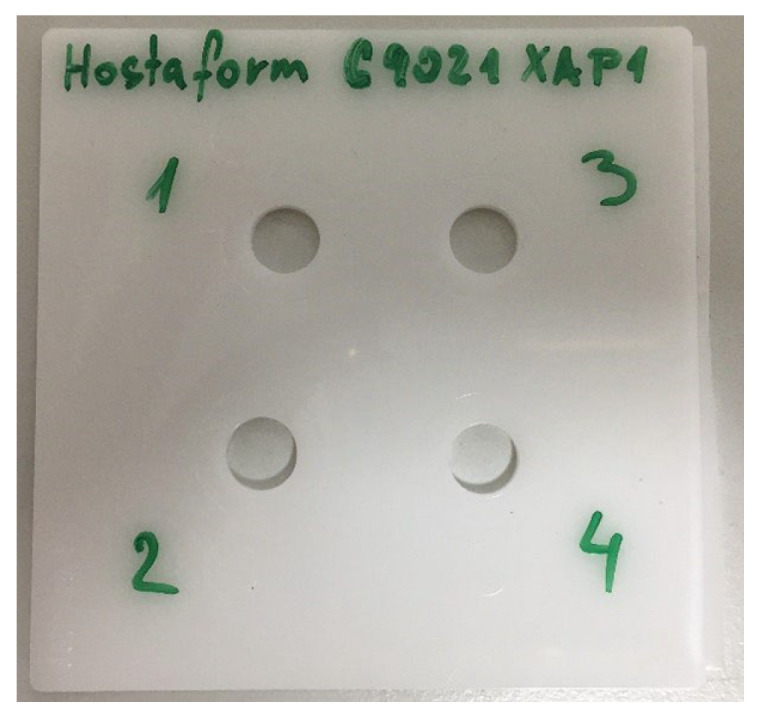
Test sample of Hostaform^®^ C9021 XAP^®^2.

Before starting the test, it was necessary to prepare plastic samples that required drilling through holes. The hole diameter was set to 8.5 mm following ISO 273 [20], which recommends a minimum hole diameter of 8.4 mm with a tolerance alignment of H12. This alignment corresponds to a tolerance range from −0 to +0.15 mm. One of the material samples prepared for testing is shown in Figure 1.

**Figure 2:**
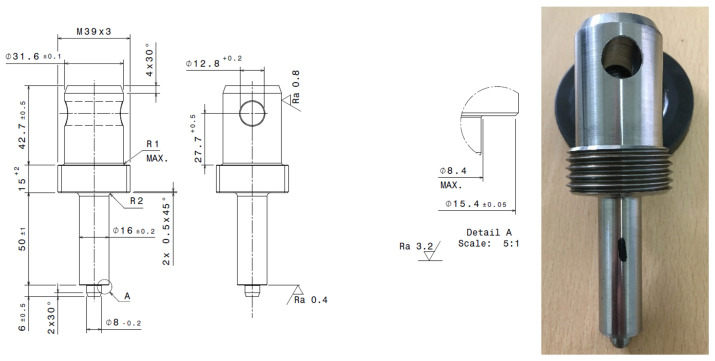
Shaft for axial force transmission during compressive stress test.

As a tool for transferring the axial force from the Instron device to the plastic sample, a specially manufactured shaft was used (see Figure 2), whose bearing surface diameter was designed according to DIN 34805-2 [21] (this standard defines the smallest diameter of the bearing surface at 15.3 mm). The shaft was manufactured by the partner company Regada, s.r.o., in Prešov, Slovakia. The shaft used had a bearing surface diameter of 15.4 ± 0.05 mm, in order to simulate the use of a bolt with a washer whose bearing surface diameter is at least 15.35 mm.

**Figure 3:**
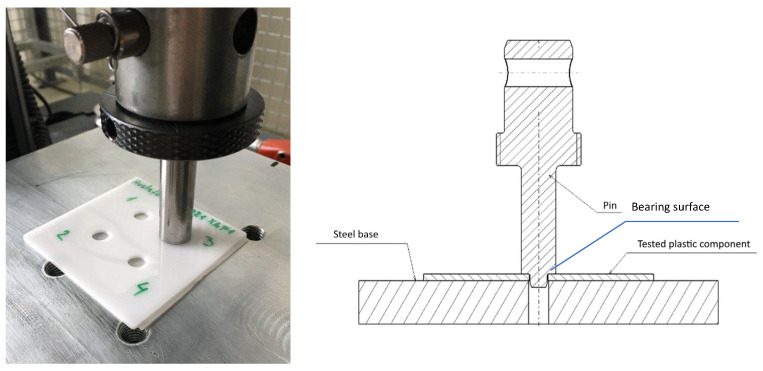
The plastic sample compressive stress test.

The course of the test was adapted for each investigated material separately (see Figure 3). The test speed was set to 50 mm/min for all samples. The end of the test was controlled based on the achieved deformation of the material in the range from 0.5 to 0.75 mm, and this parameter was adjusted depending on the specific mechanical properties of the individual materials. After reaching the specified deformation, the machine automatically stopped and returned to the starting position. The test sample was placed on a steel base with a drilled hole 8.2 mm in diameter. The shaft was slowly adjusted to touch the plastic sample. Subsequently, a preload axial force ranging from 30 to 50 N was applied (as the samples were not perfectly straight, the test began with preload). After starting the test, the device stopped when the specified deformation was reached and then returned to the starting position.

The ABS material supplied by our partner company was the first to be tested. Since the exact trade name of this material was not known and the official tabular values of the tensile strength were also missing, it was necessary to conduct the tensile test. Based on the tests carried out in the partner company, the tensile strength limit was set at approximately 48 MPa, with the measured values ranging from 48.08 MPa to 49.77 MPa. From the tests, the mean value of the yield strength under compressive stress for ABS material was calculated at the level of 99.69 MPa (see Table 1).

The 3σ statistical approach was used to determine the compressive stress limit value, which means that three times the standard deviation was subtracted from the mean value. The resulting compressive stress limit value for ABS was 86.051 MPa, which is the lower limit below which most of the measured values should not drop (only 0.3% of the values could be lower). The 3σ value in this context represents a statistical variance, with σ denoting the standard deviation. The 3σ value determines the range in which 99.7% of all measured values are located under a normal (Gaussian) distribution. This statistical methodology is used in material testing to prevent material overload, for example, during bolt tightening.

**Figure 4:**
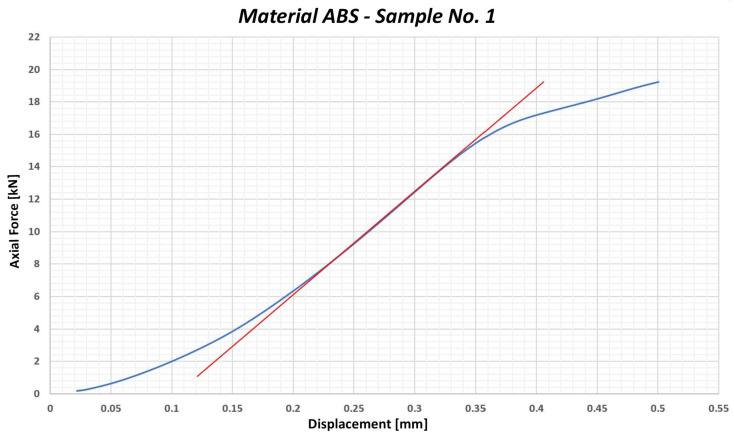
Graphical representation of the course of testing the sample no. 1—ABS material.

Based on this calculation, it is necessary to ensure that the value of 86 MPa, which represents the maximum admissible compressive stress for this material, is not exceeded during the tightening process. The graph of the testing is shown in Figure 4. The blue curve represents the dependence of the axial force on the deformation, while the red line represents the linear deformation course in the elastic mode. If the blue curve copies the direction of the red line, we are talking about elastic deformation, which means that the material returns to its original state after it is relieved. At the point where the blue curve begins to depart from the red line, the limit value of the axial force is exceeded, which the material can still transmit without suffering plastic deformation. This approach was chosen because it complies with the methodology used in testing the maximum torque and yield strength that the bolt is able to transmit. Such a procedure makes it possible to identify the point where the material or component passes from the elastic to the plastic area, which is crucial in determining its mechanical properties.

The test results provide valuable data on the mechanical properties of the ABS material, which are important for its application in industrial processes.

**Figure 5:**
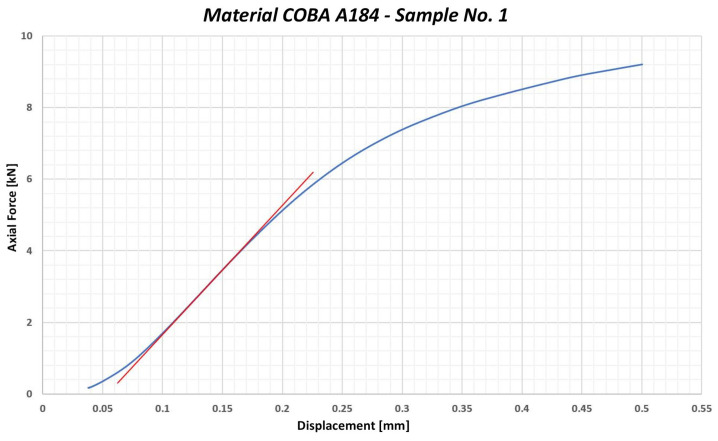
Graphical representation of the course of testing the sample no. 1—PP01 COBA A184.

Another thermoplastic polymer tested was the polypropylene PP01 COBA A184. It is a material that is known for its excellent chemical resistance, lightness and strength. The yield strength value stated in the material data sheet is 18 MPa. The average yield strength limit value under compression was measured at 32.48 MPa. Furthermore, when calculating the limit values, the statistical concept of 3σ was applied to determine the lower or upper limit, which the measured values are unlikely to exceed. In this case, when measuring the yield strength under compression, 3σ is subtracted from the average value measured (32.48 MPa), which determines the lower limit value (26.437 MPa). This means that, in 99.7% of cases, it is very likely that the compressive yield strength value will not exceed this limit value (26.437 MPa). This is the material with the lowest yield strength under compression that was used in testing. The ratio between tensile yield strength and compressive yield strength is 1.47. This means that the PP COBA A184 is almost half as strong under compressive stress than under tension stress and we should not exceed 26 MPa during the tightening process of the washer bolt. Figure 5 shows a graphical representation of the course of the test conducted with PP01 COBA A184.

**Figure 6:**
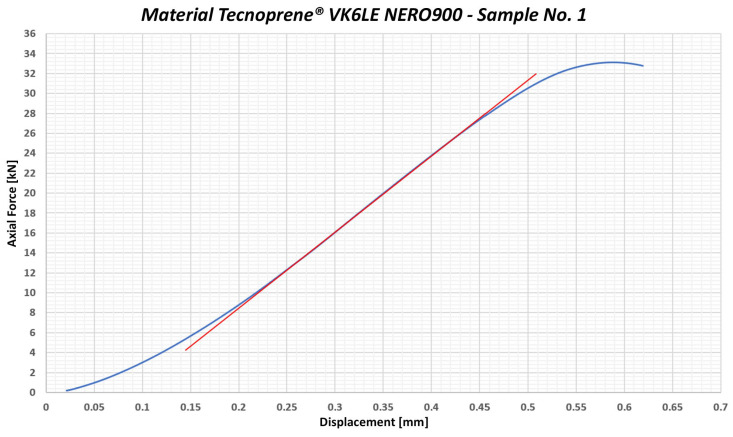
Graphical representation of the course of testing the sample no. 1—Tecnoprene^®^ VK6LE NERO900.

The third group of samples was tested using Tecnoprene^®^ VK6LE NERO900 (Celanese Corporation, Irving, TX, USA) which belongs to the category of elastomers. The graphical record of the testing process for sample no. 1—the material Tecnoprene^®^ VK6LE NERO900—is shown in Figure 6. This flexible and technically advanced material is characterized by high resistance to various chemicals, temperature changes and mechanical wear, which makes it ideal for various applications in the automotive industry.

Due to its elasticity and resistance to oils and coolants, Tecnoprene^®^ VK6LE NERO900 is often used to produce gaskets that prevent fluid leaks from designated areas in cars. In addition, it is also applied in the manufacture of housings for various electrical and mechanical components, while providing protection against external influences. This material has the ability to effectively absorb vibrations and noise, thus increasing comfort in the interior of the vehicle. According to the material data sheet, Tecnoprene^®^ VK6LE NERO900 has an ultimate strength of 85 MPa, which makes it a relatively strong material containing 30% glass fibers. The average value of the yield strength under compression was determined to be 177.33 MPa. Having accounted for the 3σ deviation, the limit value of the yield strength under compression reached 150.233 MPa. This means that the maximum permissible local stress during the screw driving process with a flat washer bolt was estimated to be 150 MPa.

For Tecnoprene^®^ VK6LE NERO900, a standard deviation of up to 1.159 kN was measured, which is approximately 5%. This significant deviation during measurement may affect the final values; therefore, the resulting limit value of the yield strength under compression is set at 150 MPa.

Since a large number of test samples of 18 thermoplastic polymers were analyzed as part of the study, Table 2 shows the final results of compressive stress tests conducted on 12 test samples for each of these analyzed materials.

Hostaform^®^ XGC10 EF XAP^®^2 is the strongest material used in the testing. With an ultimate strength (stress at break) of 110 MPa stipulated in the material data sheet, and a yield strength under compression of 180 MPa, it ranks first in terms of compression tolerance capacity of the materials tested. It is a POM material with 10% glass fiber content. In the automotive industry, it is mainly used for the production of components that require high strength and durability, such as engine covers, brackets, holders and various fasteners. Thanks to its low weight and high mechanical properties, it contributes to reducing the weight of vehicles and thus improving fuel efficiency.

Ultramid^®^ B3WZ1 HP SW805 belongs to the group of polyamides with the additional designation I. The letter I refers to the material to which modifiers have been added in the production process to increase the toughness of the material, in order to prevent damage or scratching of the plastic component during a fall or at lower temperatures due to embrittlement. Adding modifiers makes the material tougher.

PA6 GF15 Celanyl^®^ B3 GF15 NC 1102 is a polyamide with 15% glass fiber content and a relatively high tensile strength. In cars, it is used for components that require high strength, heat resistance and good mechanical properties. Its typical applications are engine covers, various brackets and holders, and interior and exterior aesthetically appealing parts.

Zytel^®^ 103HSL BKB080 is a thermally stabilized, lubricated polyamide 66 (PA66) with high mechanical strength, excellent balance between stiffness and toughness, and good properties at high temperatures. This material also has good resistance to abrasion and chemicals, making it suitable for demanding applications in the automotive, furniture, home appliances, sports equipment and construction industries.

Ultramid^®^ A3WG6 is a 30% glass fiber-reinforced polyamide 66 (PA66) that is designed for injection molding. This material is characterized by high rigidity, strength and dimensional stability, which makes it ideal for demanding applications such as machine components and covers. It also has excellent resistance to heat and chemicals.

Sabic^®^ PP 56M10 is a block copolymer of polypropylene designed for injection molding. This material is characterized by high impact resistance, even at low temperatures, and high rigidity. It is suitable for the production of buckets, containers, crates, boxes and various automotive components, especially battery packaging.

Hostaform^®^ C 13021 XAP^®^ 2 is a polyoxymethylene copolymer (POM) designed for injection molding. This material is characterized by medium creep and low emissions, which makes it ideal for use in car interiors.

Shinite^®^ D201NA is a polybutylene terephthalate (PBT) designed for injection molding. This material is characterized by excellent electrical properties and workability. It is used in a wide range of applications, including computer components, home appliances and automotive connectors.

Ultramid^®^ B3WG6 is a 30% glass fiber-reinforced polyamide 6 (PA6) designed for injection molding. This material is thermally stabilized and has excellent mechanical properties, making it suitable for various demanding applications. In the case of car structures, this material is often used for intake manifolds and pedals.

The high-temperature polyamide (PA46) Stanyl^®^ TW341 offers excellent wear and friction properties, along with exceptional creep resistance, strength, stiffness and fatigue resistance, especially at high temperatures.

Zytel^®^ MT409AHS BK010 is a material from the PA66-I polyamide group. Similarly to Ultramid^®^ B3WZ1 HP SW805, it is impact modified, i.e., it is resistant to impact and abrasion. This material is characterized by good stiffness, improved strength at the joints, a high-quality surface and excellent workability.

## 5. Testing of Material Samples—Stress Test Involving Axial Force and Torque (Combined Load)

The stress test under a combination of axial force and torque has been designed to simulate the bolted joint as closely as possible. A cylindrical head bolt was used for testing, as set out in ISO 14579 [22] or DIN 912 [23], with strength class of 12.9, according to ISO 898-1 [24]. For the purpose of the test, a standard hexagon nut with a flange according to DIN EN 1661 [25] or DIN 6923 [26], in the strength class 8 according to ISO 898-2 [27], was used.

If the matrix was used more than twice, the values of the measured axial force decreased rapidly. For this reason, the nut was replaced after each conducted test. The reason may be damage to the surface treatment and a subsequent increase in the friction coefficient. The bolt did not show any wear, so it was not replaced during testing. For testing, an axial force sensor from from the German company Hottinger Baldwin Messtechnik (type KMR+) was used, designed for a bolt size of 8 mm, with a load capacity of 40,000 N.

The samples of plastic parts were purchased from an external supplier. When selecting them, the manufacturing technology was taken into account, which affects the final structure, density, homogeneity, and internal stresses of the material, which in turn influences its mechanical properties. The most commonly used technology for producing plastic parts in the automotive industry is plastic injection molding. This criterion was decisive in the selection and subsequent preparation of the analyzed samples.

**Figure 7:**
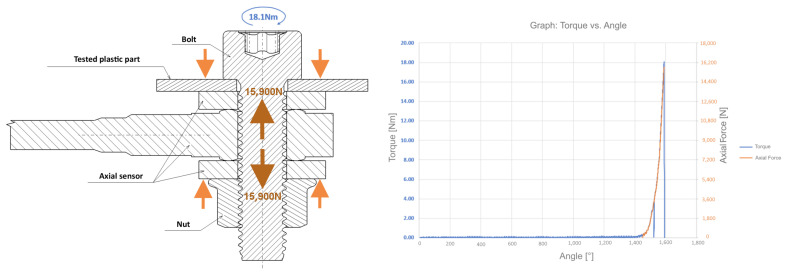
Diagram of the testing under implementation of axial force and torque.

Before the start of the test, holes with a diameter of 8.5 mm were drilled into the plastic part, while the outer diameter of the bearing surface was 12.33 mm, in accordance with the requirements of the relevant standard. The holes were drilled so that the distance between their axes was approximately four times the diameter of the hole, which is a common arrangement in technical applications. The axial distance of the drilled holes in the prepared samples was 34 mm. These dimensions were subsequently used in the calculations of the stress applied. During the test, two basic values were measured: the axial force recorded by the axial force sensor and the torque detected by the electric torque wrench ETV ST61-30-10. This application is illustrated with arrows in the diagram (Figure 7). Torque refers to the tightening moment (on Figure 7, marked in blue on the left.). Axial force refers to the axial force generated as a result of a specific tightening torque (on Figure 7, shown on the left in orange). The graph shown in Figure 7 follows the applicable standard and represents the recommended tightening torque for metallic components being joined. On the contact surface, pressure is generated, and its limiting values were determined through experimental measurements.

The defined tightening moment generates an axial force in the joint ranging from 14,700 to 17,900 N. For a contact area of 52.93 mm^2^, this corresponds to a bearing pressure under the bolt head of 277.74 to 338.2 MPa. This means that, if a plastic component with a compressive yield strength lower than 338.2 MPa is used, plastic deformation or even failure of the plastic component would occur.

To prevent this, it is essential to reduce the pressure under the bolt head to ensure the plastic component is only subjected to stress within its allowable limits. Pressure reduction can be achieved by decreasing the tightening torque. However, the range of the tightening moment is limited, as it is necessary to maintain a torque value that ensures the clamping force does not drop below a level that would lead to the loosening of the bolted joint. The tightening speed was the same for all test samples and set to 50 rpm to ensure consistency of results.

**Figure 8:**
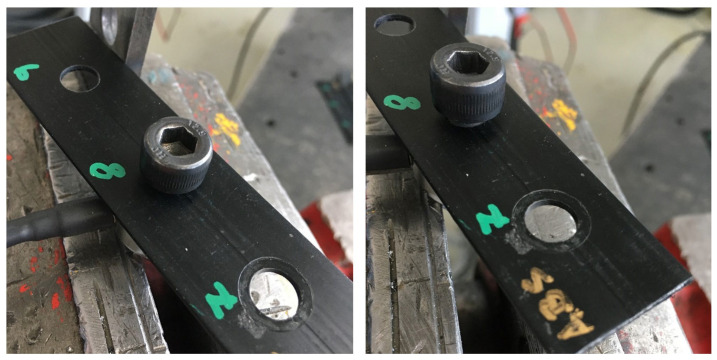
The stress test involving axial force and torque.

**Figure 9:**
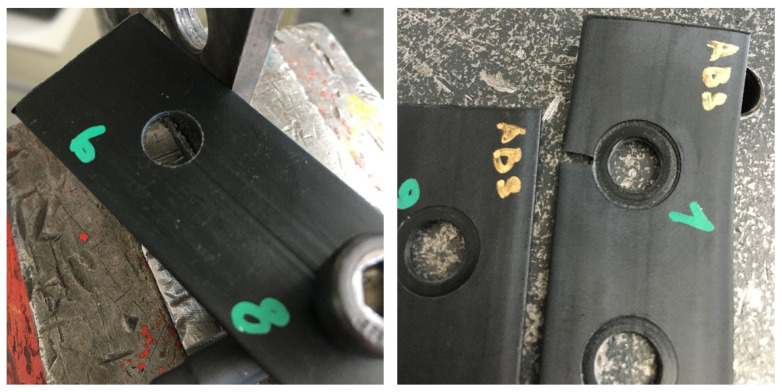
Detail of the sample before the test and damage to the sample.

This approach has ensured accurate and repeatable results that allow for the assessment of the plastic materials’ properties in conditions close to real use in industrial practice. Figure 8 shows photos of the tested sample of the ABS material. The numbers displayed on the analyzed material represent the order of the tested sample for the axial force and torque test. In Figure 9, there is observable damage to the test sample (ABS material) due to high torque. Of course, the distance between the holes or between a hole and the edge can significantly affect the strength of the joints, as insufficient distances lead to higher stress concentrations. This concentration can result in the formation of cracks. In our experiment, the dimensions of the samples were chosen to reflect real technical applications in the product range of our partner and to ensure the representativeness of the results.

**Figure 10:**
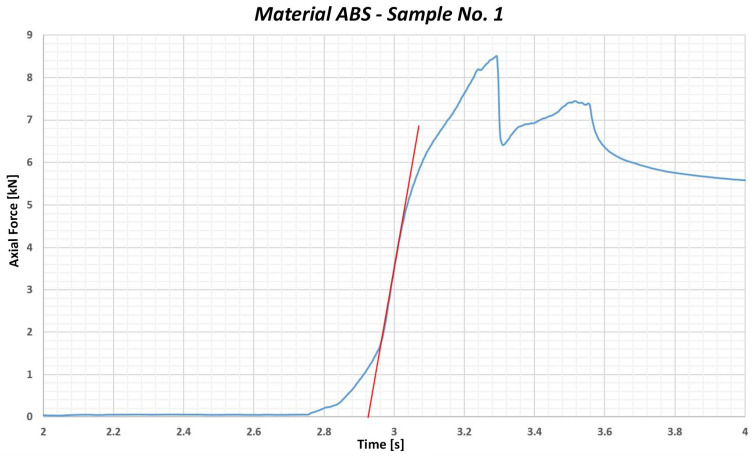
Graphical representation of testing the sample no. 1 of ABS material—compressive stress and torsion test (combined load).

As when we tested the material samples under compressive stress, a total of 12 samples were tested in the course of this testing, involving a combined load. Sample no. 12 showed a disproportionately high axial force value (6.5 kN) and was, therefore, excluded from the analysis. The abnormally high value measured in sample 12 could have been caused by several factors; e.g., the sample material could have had internal defects or inhomogeneities, such as inclusions or microcracks, that altered its mechanical properties. These deficiencies may have affected the stress distribution and caused anomalous results. The sample may also have been incorrectly prepared, e.g., if its geometry was not accurate (incorrect hole size, thickness, or deformation during preparation), the stresses during the test may have been incorrectly distributed. This would increase the axial force required for sample failure. Last but not least, the material of sample no. 12 could have been subjected to different heat treatment, higher humidity or other environmental influences that could have increased its strength compared to other samples. The calculations were thus made on the basis of testing the remaining 11 samples. The average yield strength value, calculated from the measured values, was determined to stand at 70.96 MPa. After taking into account the standard deviation of 3σ, the limit value of the yield strength was corrected to 59.01 MPa (see Table 3 and Figure 10). Based on these results, the maximum compressive stress limit value for the standard screw driving process was set at 59 MPa, which is the safe limit for this material.

**Figure 11:**
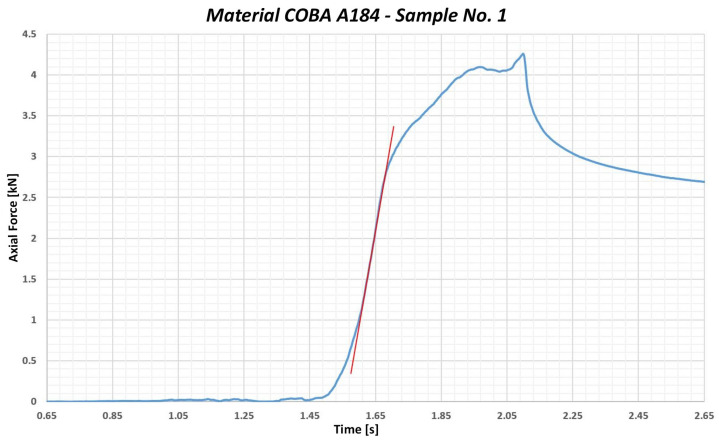
Graphical representation of testing the sample no. 1 of the PP01 COBA A184—stress and torsion test (combined load).

Similarly to the stress tests case, another material tested was polypropylene PP01 COBA A184, with a declared yield strength of 18 MPa. Figure 11 shows a graph of the testing progression for sample no. 1 of the material PP01 COBA A184 during the stress test involving axial force and torque. The average value of the yield strength under a combined load was determined to be 33.71 MPa. After including the 3σ standard deviation, the resulting yield strength limit under a combined load was calculated to be 19.27 MPa. During the tightening process of the bolt without a washer, it is recommended not to exceed the value of 19 MPa as the maximum compressive stress value admissible.

**Figure 12:**
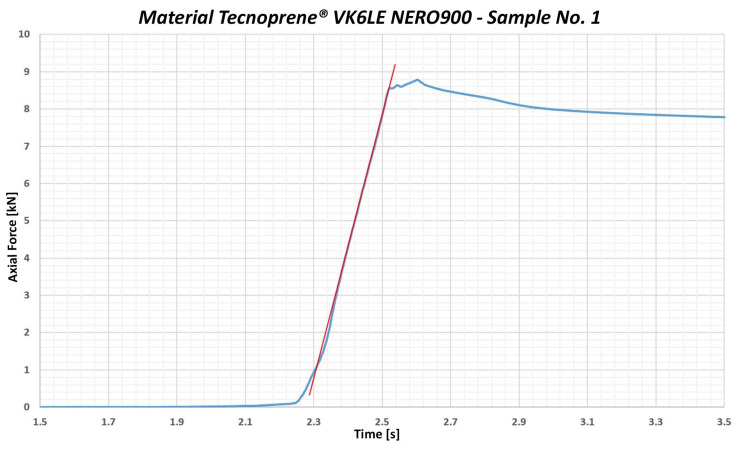
Graphical representation of testing the sample no. 1 of Tecnoprene^®^ VK6LE NERO900—stress test involving axial force and torque.

The third group of samples comprised polypropylene Tecnoprene^®^ VK6LE NERO900. It is a polypropylene containing 30% glass fiber. The material has a defined ultimate strength (stress at break) of 85 MPa. From the measured values, the yield strength under a combined load was determined under the stress of 143.03 MPa. With a standard deviation of σ = 9.563 MPa and accounting for 3σ, the limit value of the yield strength under a combined load is 114.342 MPa. The maximum compressive stress value is 114 MPa (see Figure 12).

Tests for all other materials were carried out in the same way. Table 4 contains a list of the results of the tests carried out in the stress test involving axial force and torque for other analyzed material samples.

## 6. Discussion

In summary, the results of the measurements of the tests carried out are shown in Table 5.

The test results show that the tensile stress limits indicated in the material data sheets are, in most cases, 5–10% lower than, and in some cases by as much as more than 50% lower than, the compressive stress limits. The ratio between these values varies, but we can assume with high probability that if, when calculating the torque in the initial phases of the bolted joint design, we use the tensile limits defined in the material data sheet as the maximum ones allowed, we will prevent deformation or damage to the plastic component.

It is possible to exceed the limit tensile value by 5 to 10% in the initial stages of bolted joint design with the occurrence of plastic deformation being unlikely. If the compressive stress on the bearing surface exceeds 110% of the tensile limit, the design needs to be modified. In the initial stages, it is possible to recommend the use of a material with a higher ultimate strength or a bolt with a washer, as the compressive stress limits for pure compression are higher (see Table 5). If changing the material would not be possible due to increased costs associated with stronger materials, the authors of this study recommend conducting physical tests. This is particularly important due to the high variability in the ratio between the tensile yield strength or ultimate strength specified in the material data sheet and yield strength under combined load limits, which ranged from 1:1 to 1:2.24 in presented study.

Based on the results of the analysis, it is possible to set the compressive stress limit as up to 150% of the tensile stress limit for the material from the POM group, with the risk of plastic deformation being minimal. In the case of using PA6, PA46 or PA66 materials, the above recommendations should be followed, because the compressive stress limit ranged from 95% to 177% of the tensile stress limit. This procedure is also recommended for materials from the PP group and other plastic groups. However, if the material used in the testing is used, we recommend following the limit values given in the summary table (Table 5).

Based on the compressive stress limits, it is possible to determine the minimum diameter of the bearing surface under the bolt head and to define the optimal tightening moment value, so as to prevent the deformation of the plastic component or loss of axial clamping force in the bolted joint, which could lead to the loosening of the joint.

## 7. Conclusions

The testing of plastic components in the automotive industry plays a key role in ensuring the quality, reliability and safety of the final products. With the boom in the use of plastics in vehicle construction, it is necessary to constantly innovate testing methods that can detect the weaknesses of materials before they are introduced into mass production. In this paper, we have analyzed possible approaches and techniques that are used in the evaluation of bolted joints of plastic parts. Based on the tests conducted, it has been shown that plastic components, especially in bolted joints, can withstand significantly higher compressive stress values than their tensile stress limit. These results provide important information for optimizing the design of bolted joints of plastic components in the automotive industry. For individual materials such as ABS, PA, PBT, POM and PP, physical testing is recommended due to the variability of the tensile stress/compressive stress limit ratio, which is crucial for the design of reliable and durable joints. If the compressive stress limit values are exceeded, adjustments in the joint design, such as the use of a washer or a material with higher strength, should be chosen to avoid deformations and thus ensure a higher reliability of the joint.

Future trends in the testing of plastic parts are likely to include an even more intensive integration of simulations and predictive models that will enable a reduction in the time needed to verify quality, speeding up the entire production process while reducing waste. Such innovations will have a positive impact not only on production efficiency, but also on the overall sustainability of the automotive industry.

## Figures and Tables

**Table 1:** Table of measured values: compressive stress test for ABS material.

Sample Number	Hole Diameter $D_{h}$ [mm]	Bearing Surface Diameter $d_{w}$ [mm]	Axial Force $F_{M}$ [kN]	Yield Strength Under Compression σ [MPa]
1	8.5	15.35	13.57	105.76
2	8.5	15.35	13.44	104.74
3	8.5	15.35	13.76	107.24
4	8.5	15.35	12.73	99.21
5	8.5	15.35	12.82	99.91
6	8.5	15.35	12.60	98.20
7	8.5	15.35	11.96	93.21
8	8.5	15.35	13.24	103.19
9	8.5	15.35	12.24	95.39
10	8.5	15.35	12.31	95.94
11	8.5	15.35	12.53	97.65
12	8.5	15.35	12.30	95.86

**Table 2:** Results of compressive stress tests conducted on 12 test samples.

Material (Regada, s.r.o., Prešov, Slovakia)	Ultimate Strength—Material Data Sheet [MPa]	Yield Strength Under Compression—Material Data Sheet [MPa]	Measured Average Yield Strength Under Compression Calculated on 12 Samples [MPa]	Yield Strength Limit under Compression After Subtracting the Standard Deviation [MPa]	Maximum Compressive Stress Limit for Washer Bolt [MPa]
Hostaform^®^ XGC10 EF XAP^®^2	110	180	177.41	166.822	166
Ultramid^®^ B3WZ1 HP SW805	50	64	62	52.83	52
PP	28	40	54.81	44.515	44
Celanyl^®^ B3 GF15 NC 1102	130	140	143.14	136.571	136
Delrin^®^ 500T NC010	52	55	160.73	154.563	154
Zytel^®^ 103HSL BKB080	85	55	96.33	89.036	89
Zytel^®^ 101F NC010	82	55	101.9	97.358	97
Ultramid^®^ A3WG6	130	190	168.36	156.384	156
Ultramid^®^ B35EG3	70	130	137.73	123.322	123
Sabic^®^ PP 56M10	35	29	49.523	37.163	37
Hostaform^®^ C 13021 XAP^®^ 2	65	64	137.841	128.024	128
Shinite^®^ D201NA	54	83	107.71	98.372	98
Ultramid^®^ B3WG6	115	180	156.38	148.32	148
Stanyl^®^ TW341	100	55	87.92	79.44	79
Zytel^®^ MT409AHS BK010	61	42	67.586	61.764	61

**Table 3:** Table of measured values: stress test involving axial force and torque (combined load) for ABS material.

Sample Number	Hole Diameter $D_{h}$ [mm]	Bearing Surface Diameter $d_{w}$ [mm]	Axial Force $F_{M}$ [kN]	Yield Strength under Combined Load σ [Mpa]
1	8.5	12.33	4.26	67.96
2	8.5	12.33	4.24	67.67
3	8.5	12.33	4.50	71.77
4	8.5	12.33	4.44	70.86
5	8.5	12.33	4.09	65.19
6	8.5	12.33	4.58	73.09
7	8.5	12.33	4.47	71.34
8	8.5	12.33	4.52	72.06
9	8.5	12.33	4.92	78.52
10	8.5	12.33	4.74	75.65
11	8.5	12.33	4.17	66.49
12	8.5	12.33	-	-

**Table 4:** Results of stress test involving axial force and torque (combined load) conducted on 12 test samples.

Material (Regada, s.r.o., Prešov, Slovakia)	Measured Average Yield Strength Calculated on 12 Samples [MPa]	Yield Strength Limit Under a Combined Load after Subtracting the Standard Deviation [MPa]	Maximum Stress Limit Under a Combined Load for Washer Bolt [MPa]
Hostaform^®^ XGC10 EF XAP^®^2	188.88	163.716	163
Ultramid^®^ B3WZ1 HP SW805	57.98	47.318	47
PP	22	46.34	30
Celanyl^®^ B3 GF15 NC 1102	144.22	1367.057	137
Delrin^®^ 500T NC010	144.45	123.456	123
Zytel^®^ 103HSL BKB080	82.86	67.170	67
Zytel^®^ 101F NC010	85.05	76.258	76
Ultramid^®^ A3WG6	179.37	146.464	146
Ultramid^®^ B35EG3	100.45	85.082	85
Sabic^®^ PP 56M10	49.91	36.523	36
Hostaform^®^ C 13021 XAP^®^ 2	120.22	105.404	105
Shinite^®^ D201NA	78.94	70.843	70
*Ultramid^®^ B3WG6*	172.29	144.528	144
*Stanyl^®^ TW341*	79.34	60.713	60
*Zytel^®^ MT409AHS BK010*	69.28	58.881	58

**Table 5:** Summary of the results of the both types of tests conducted.

Group	Sub-Group	Material Specification	Additional Material Information	*σ*_t_ in Tension	Value *σ*_t_ [MPa]	Yield Strength Under Compression *σ*_T_ [MPa]	Ratio *σ*_T_/*σ*_t_	Yield Strength Under a Combined Load *σ*_TK_ [MPa]	Ratio *σ*_TK_/*σ*_t_
ABS	ABS	ABS	-	Stress at break	48	86	1.79	59	1.23
PA	PA46	Stanyl^®^ TW341	Thermally stabilized, lubricated	Stress at break	55	79	1.44	60	1.10
	PA6-I	Ultramid^®^ B3WZ1 HP SW805	Thermally stabilized	Stress at break	50	52	1.06	47	0.95
	PA6 GF15	Ultramid^®^ B35EG3	-	Stress at break	70	123	1.76	85	1.22
	PA6 GF15	Celanyl^®^ B3 GF15 1102	Values of tensile strength for moisture content <0.2%	Stress at break	130	136	1.05	137	1.05
	PA6 GF30	Ultramid B3WG6	-	Stress at break	115	148	1.29	144	1.26
	PA66	Zytel^®^ 103HSL BKB080	Unhardened, thermally stabilized	Yield strength	55	89	1.62	67	1.22
	PA66	Zytel^®^ 101F NC010	Unhardened	Yield strength	55	97	1.77	76	1.39
	PA66-I	Zytel^®^ MT409AHS BK010	Cured, thermally stabilized	Yield strength	42	61	1.47	58	1.40
	PA66 GF30	Ultramid^®^ A3WG6	-	Stress at break	130	156	1.20	146	1.13
PBT	PBT	Shinite^®^ D201G20BK	-	Stress at break	95	165	1.74	126	1.33
POM	POM	Hostaform^®^ C 13021 XAP^®^2	-	Yield strength	64	128	2.00	105	1.65
	POM-I	Delrin^®^ 500T	Cured	Stress at break	55	154	2.80	123	2.24
	POM GF10	Hostaform^®^ XGC10 EF XAP^®^2	-	Stress at break	110	166	1.52	163	1.49
PP	PP01	COBA A184	-	Yield strength	18	26	1.47	19	1.07
	PP	PP	-	Stress at break	22	44	1.98	30	1.37
	PP	Sabic^®^ PP 56M10	-	Stress at break	27	37	1.38	36	1.35
	PP GF30	Tecnoprene^®^ VK6LE NERO900	-	Stress at break	85	150	1.77	114	1.35

## Data Availability

The data presented in this study are available on request from the corresponding author. The data are not publicly available due to restrictions on funding sources.

## References

[1] Taub A., De Moor E., Luo A., Matlock D.K., Speer J.G., Vaidya U. (2019). Materials for Automotive Lightweighting. Ann. Rev. Mater. Res..

[2] Nam J.Y., Kim D.W., Oh J.H. (2022). Investigation of the fastening behavior of self-tapping plastic joints with various supporting ribs. J. Manuf. Process..

[3] Cumbicus W.E., Estrems M., Arizmendi M., Jiménez A. (2023). Analysis of self-tapping screw joints in fibre glass reinforced PEI polymer used in the automotive industry. Int. J. Adv. Manuf. Technol..

[4] Nekrasov S., Zhyhylii D., Dovhopolov A., Altin Karatas M. (2021). Research on the manufacture and strength of the innovative joint of FRP machine parts. J. Manuf. Process..

[5] Martinsen K., Hu S., Carlson B. (2015). Joining of dissimilar materials. Cirp Ann..

[6] Shuvaev V. (2022). Control of the tightening of threaded joints during ultrasonic assembly by assessing the transition to the zone of plastic deformations. AIP Conf. Proc..

[7] Bazaras Ž., Leonavičius M., Lukoševičius V., Raslavičius L. (2021). Assessment of the Durability of Threaded Joints. Appl. Sci..

[8] Li H., Guo F., Han C., Su W., Wen S. (2024). Mechanical Properties of Carbon Fiber-Reinforced Plastic with Two Types of Bolted Connections at Low Temperatures. Polymers.

[9] McCarthy M.A., Lawlor V.P., Stanley W.F., McCarthy C.T. (2002). Bolt–hole Clearance Effects and Strength Criteria in Single-bolt, Single-lap, Composite Bolted Joints. Compos. Sci. Technol..

[10] El-Sisi A., Hassanin A., Alsharari F., Galustanian N., Salim H. (2022). Failure Behavior of Composite Bolted Joints: Review. CivilEng.

[11] Galińska A. (2020). Mechanical Joining of Fibre Reinforced Polymer Composites to Metals—A Review. Part I: Bolted Joining. Polymers.

[12] Liu Q., Song P., Yan J., Sun M., Wang K., Wang Y., Qing X. (2024). In Situ Monitoring of Failure Modes in Composite Bolted Joint Structures Using CB/CNT Piezoresistive Sensor. IEEE Sens. J..

[13] Tobalina-Baldeon D., Sanz-Adán F., Martinez-Calvo M., Gómez C., Sanz-Pena I., Cavas F. (2021). Feasibility Analysis of Bolted Joints with Composite Fibre-Reinforced Thermoplastics. Polymers.

[14] Liu F., Xie M., Ji Y., Zhou M. (2020). Progressive fatigue damage analysis of composite bolted joint using equivalent stress model. Sci. Prog..

[15] (2019). Metallic Materials—Tensile Testing—Part 1: Method of Test at Room Temperature.

[16] Lampman S. (2003). Characterization and Failure Analysis of Plastics.

[17] Du Y., Keller T., Zhu Y., Wei P., Wang Y., Xiong J. (2023). Mechanical behavior and failure of carbon fiber-reinforced composite sandwich structure inspired by curved-crease origami. Compos. Struct.

[18] NYLOK PRECOTE® 85. Macomb Township, Michigan: NYLOK. https://nylok.com/pre-applied-processes/chemicallocking/precote-85/.

[19] (2015). Systematic Calculation of Highly Stressed Bolted Joints—Joints with One Cylindrical Bolt.

[20] (1979). Fasteners—Clearance Holes for Bolts and Screws.

[21] (2018). Button Head Screws—Part 2: SCREWS with Collar and Driving Feature Hexalobular Socket.

[22] (2011). Hexalobular Socket Head Cap Screws.

[23] DIN 912. https://www.aramfix.com/content/files/d912caill/datasheet%20din%20912.pdf.

[24] (2013). Mechanical Properties of Fasteners Made of Carbon Steel and Alloy Steel. Part 1: Bolts, Screws and Studs with Specified Property Classes—Coarse Thread and Fine Pitch Thread.

[25] (1996). Hexagon Nuts with Flange.

[26] DIN 6923. https://www.fasteners.eu/standards/din/6923/?utm_source=chatgpt.com#google_vignette.

[27] (2022). Fasteners—Mechanical Properties of Fasteners Made of Carbon Steel and Alloy Steel. Part 2: Nuts with Specified Property Classes.

